# BatchSVG: identifying batch-biased genes in the application of spatially variable gene detection

**DOI:** 10.1093/bioinformatics/btag522

**Published:** 2026-07-16

**Authors:** Kinnary Shah, Christine Hou, Jacqueline R Thompson, Stephanie C Hicks

**Affiliations:** Department of Biostatistics, Johns Hopkins Bloomberg School of Public Health, Baltimore, MD 21205, United States; Department of Biostatistics, Johns Hopkins Bloomberg School of Public Health, Baltimore, MD 21205, United States; Department of Biostatistics and Medical Informatics, University of Wisconsin-Madison, Madison, WI 53726, United States; Department of Biostatistics, Johns Hopkins Bloomberg School of Public Health, Baltimore, MD 21205, United States; Department of Biostatistics, Johns Hopkins Bloomberg School of Public Health, Baltimore, MD 21205, United States; Department of Biomedical Engineering, Johns Hopkins University, Baltimore, MD 21218, United States; Kavli Neuroscience Discovery Institute, Johns Hopkins University, Baltimore, MD 21205, United States; Center for Computational Biology, Johns Hopkins University, Baltimore, MD 21211, United States; Malone Center for Engineering in Healthcare, Johns Hopkins University, Baltimore, MD 21218, United States

## Abstract

**Summary:**

A standard task in the analysis of spatially resolved transcriptomics (SRT) data is to identify spatially variable genes (SVGs). This is most commonly done within one tissue section at a time because the spatial relationships between the tissue sections are typically unknown. However, large-scale spatial atlases are being generated, for example across hundreds of donors, where the goal is to identify a common set of SVGs to use for downstream analyses. One challenge is how to identify and remove SVGs that are associated with a known bias or technical artifact, such as the slide, which can lead to poor performance in downstream analyses, such as spatial domain detection. Here, we introduce BatchSVG, a tool to identify batch-biased genes SVGs. Our approach compares the rank of per-gene deviance under a binomial model (i) with and (ii) without including a covariate in the model that is associated with the known bias or technical artifact. If the rank of a gene changes significantly between these models, then we infer that this gene is likely associated with the bias or technical artifact and should be removed from the downstream analyses. We consider two SRT datasets and show how our model can improve the results of downstream analyses.

**Availability and implementation:**

The BatchSVG package is freely available at https://bioconductor.org/packages/BatchSVG, and the code to reproduce the figures is publicly available at https://github.com/kinnaryshah/BatchSVG-analyses.

## 1 Introduction

Recent advances have led to the development of spatially resolved transcriptomics (SRT) technologies in which genes can be measured in a 2D spatial context from individual tissue sections (Rao *et al.* 2021). A common data analysis task is identifying spatially variable genes (SVGs), which are input into downstream analyses, such as unsupervised clustering algorithms to identify spatial domains (Liu *et al.* 2023) and enhance the identification of spatial domains over non-spatial feature selection (Li *et al.* 2023, Chen *et al.* 2025). Current SVG detection methods do not accommodate multi-sample datasets due to the lack of formal spatial relationships between tissue sections. Unlike non-spatial feature selection methods that model batch effects that are present in experiments with complex study designs (e.g. donor identity, batch), it is challenging to identify SVGs that are associated with technical artifacts (Yan *et al.* 2025). Batch correction methods typically result in non-count embeddings; therefore, these methods generally are not applied before SVG detection. Through our analyses of whole-transcriptome SRT data, we found that batch-biased SVGs can confound clustering results in small, multi-sample experiments.

To address this challenge, we developed BatchSVG, a method to identify SVGs that are associated with unwanted technical variation. BatchSVG builds upon the binomial deviance model (Townes *et al.* 2020) and applies a data-driven thresholding approach to refine SVG features used as input to downstream analyses. We implemented BatchSVG as an R/Bioconductor package to facilitate accessibility and integration into existing SRT analysis pipelines and to ensure compatibility with the SpatialExperiment framework (Righelli *et al.* 2022).

## 2 Materials and methods

### 2.1 Overview of BatchSVG methodology

For a given set of tissue sections t∈(1,…,T), we calculate the per-gene residual deviance under a binomial model as


$$D_{j}=2\sum_{i}\left[y_{ij}\;\text{log}\;\left(\frac{y_{ij}}{n_{i}{\hat{\pi }}_{j}}\right)+(n_{i}-y_{ij})\;\text{log}\;\left(\frac{\left(n_{i}-y_{ij}\right)}{\left(1-{\hat{\pi }}_{j}\right)}\right)\right];$$

$y_{ij}$ is the observed count for gene *j* in cell *i*, $n_{i}$ is the total number of UMIs in cell *i*, and $\pi_{ij}$ is the probability that a UMI comes from gene j in cell i (Townes *et al.* 2020). We consider all spatial coordinates across tissue sections as independent observations, so the spatial information is not incorporated. These models are fit per gene and the residual deviance is calculated for gene i∈(1,…,n). Generally, a higher per-gene deviance $d_{i}$ suggests that the gene’s expression is more likely to be biologically meaningful.

To assess the impact of a batch effect in SRT data, we fit a binomial model per gene (i) with and (ii) without including a covariate in the model that is associated with the batch effect. In model (ii), with a chosen batch covariate, the dispersion is modeled in a batch-specific way. A reduction in deviance after accounting for the batch covariate indicates that the batch explains a portion of the variation in gene expression that was previously attributed to biological differences. Using this model output above, we define $d_{i,\;\text{batch}\;\text{name}}$ and $d_{i,\;\text{default}}$ as the residual deviance for gene *i* using a binomial model with and without the batch effect, respectively. Finally, we calculate a per-gene relative change in deviance (RCD) as $RCD_{i}=\frac{d_{i,\;\text{default}}-d_{i,\;\text{batch}\;\text{name}}}{d_{i,\;\text{batch}\;\text{name}}}$.

In addition to the residual deviance itself, we also consider the ranks of the residual deviances, where top-ranked genes have the largest residual deviance. Here, an increase in rank when including the batch covariate indicates that the relative importance of the gene is diminished once the batch variable is accounted for. Therefore, we also evaluate the rank difference (RD), which is defined as $RD_{i}=r_{i,\;\text{batch}\;\text{name}}-r_{i,\;\text{default}}$, where $r_{i,\;\text{default}}$ and $r_{i,\;\text{batch}\;\text{name}}$ are the per-gene ranks when the binomial deviance model is run with and without the batch variable, respectively.

The continuous nature of RCD and RD complicates the determination of a specific threshold for identifying batch-biased genes. To this end, we developed a data-driven thresholding approach based on the number of standard deviations (nSD) of RCD and RD. The nSD for per-gene RCD is defined as $nSD_{i,\mathrm{RCD}}=\frac{RCD_{i}-\bar{\mathrm{RCD}}}{SD_{\mathrm{RCD}}}$, where $\bar{\mathrm{RCD}}=\frac{1}{n}\sum_{i=1}^{n}RCD_{i}$ and $SD_{\mathrm{RCD}}=\sqrt{\frac{1}{n-1}\sum_{i=1}^{n}{\left(RCD_{i}-\bar{\mathrm{RCD}}\right)}^{2}}.$ The nSD for per-gene RD is computed using the same formula, defined as $nSD_{i,RD}=\frac{RD_{i}-\bar{RD}}{SD_{RD}}$, where $\bar{RD}=\frac{1}{n}\sum_{i=1}^{n}RD_{i}$ and $SD_{RD}=\sqrt{\frac{1}{n-1}\sum_{i=1}^{n}{\left(RD_{i}-\bar{RD}\right)}^{2}}.$ We leverage this to establish adaptive thresholds for dataset-specific variability in RCD and RD. To identify batch-biased genes, users should tune these parameters based on dataset characteristics, choosing as conservative a threshold that they determine appropriate.

**Figure 1:**
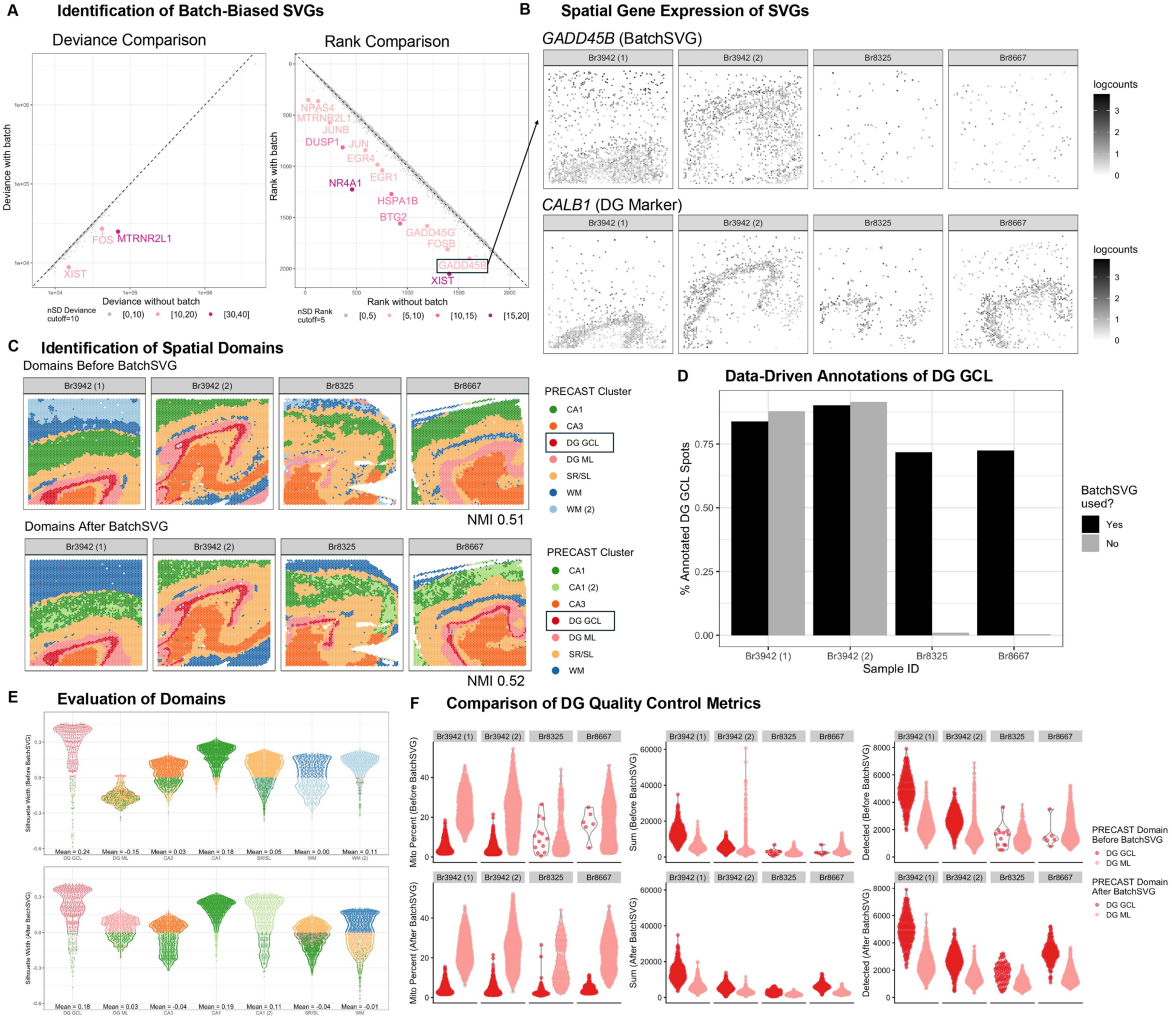
(A) Left scatterplot displays the change in deviance for each gene. The *x*-axis is deviance calculated without any covariates. The *y*-axis is deviance calculated with sample as the covariate. Color represents the number of standard deviations of relative change in deviance. The right scatterplot is similar, substituting rank for deviance. (B) Top spot plot shows the log-normalized gene expression for *GADD45B*, the batch-biased SVG boxed in (A). Bottom spot plot shows the log-normalized gene expression for *CALB1*, a dentate gyrus (DG) marker. (C) Top spot plot shows the clusters derived from PRECAST when all SVGs were used as input. Bottom spot plot shows the clusters derived from PRECAST when the batch-biased SVGs were excluded from the input SVG list. Color represents the following labels—CA: cornu ammonis, DG GCL: dentate gyrus granule cell layer, DG ML: dentate gyrus molecular layer, SR/SL: stratum lucidum and stratum radiatum, WM: white matter. Text displays the normalized mutual information (NMI) for each set of domains compared to previous annotations from the paper. (D) Bar plot displays the percentage of DG GCL spots from the previous annotations that were called as DG GCL spots in either set of PRECAST domains. Color represents domains derived from PRECAST when the batch-biased SVGs were included or excluded from the input SVG list. (E) Top violin plots display silhouette scores for domains derived from PRECAST when all SVGs were used as input. Bottom violin plots display silhouette scores for domains derived from PRECAST when the batch-biased SVGs were excluded. Color represents the closest domain. (F) Violin plots display quality control metrics for domains derived from PRECAST when the batch-biased SVGs were included (top row) or excluded (bottom row) from the input SVG list. Left row displays mitochondrial percent, middle row displays total number of UMIs, and right row displays number of detected genes. Color represents the domain.

We implemented our method in the BatchSVG R/Bioconductor package, which provides a framework to efficiently identify SVGs impacted by batch effects, such as sample, slide, and sex. The package is designed to work with SpatialExperiment objects (Righelli *et al.* 2022). The workflow (Fig. S1, available as supplementary data at *Bioinformatics* online) includes feature selection, visualization, and extraction of batch-biased features. Please see the vignette for further details (https://bioconductor.org/packages/BatchSVG). In particular, the scatterplots from the svg_nSD() function should be utilized to identify cutoff thresholds for nSD of RCD and RD. SVGs that are further away from the *y = x* line are more likely to be batch-biased (Fig. 1A).

### 2.2 Evaluation of BatchSVG’s downstream impacts

To evaluate BatchSVG, we applied PRECAST (Liu *et al.* 2023) for clustering based on the original feature set and the refined feature set after removing batch-biased SVGs. PRECAST simultaneously accounts for batch effects and calculates spatially aware clusters. Clusters were evaluated for consistency with known biology based on marker gene expression and consistency between individual samples based on percent abundance. Cluster agreement and similarity were assessed with normalized mutual information (NMI) and silhouette scores. Common quality control (QC) metrics were evaluated for each cluster from the original feature set and the refined feature set to examine if differences in clustering outcomes may be addressed by stricter upstream QC filters.

## 3 Results

### 3.1 Dentate gyrus identification in human hippocampus

This dataset was generated from tissue sections of the human hippocampus across ten adult neurotypical donors, using 10× Genomics Visium (*n *= 36 capture areas) (Thompson *et al.* 2024). It comprises nine slides with four samples per slide, of which we selected a subset of four samples from the raw SRT dataset. We leveraged the nnSVG-based SVG selection process from the original manuscript (Weber *et al.* 2023, Thompson *et al.* 2024) to include the top 2000 ranked features and only genes that appear in more than one sample. With this approach, we obtained 2082 SVGs across the four samples. Using BatchSVG with sample as the batch, nSD RCD as 10, and nSD RD as 5, we identified 15 batch-biased SVGs (Fig. 1A; Table S1, available as supplementary data at *Bioinformatics* online). The spatial expression plots confirmed that these features are spatially variable and batch-biased (Fig. 1B; Fig. S2, available as supplementary data at *Bioinformatics* online). We defined the refined feature set as the 2082 original SVGs minus the 15 batch-biased SVGs to get 2067 SVGs. Utilizing *k *= 7 clusters, we found that clusters based on the original feature set and the refined feature set both described many known domains in the human hippocampus (Fig. 1C; Fig. S3, available as supplementary data at *Bioinformatics* online). However, we highlight the red dentate gyrus granule cell layer (DG GCL) domains, which are outlined by the *CALB1* marker gene (Fig. 1B), and are missing from two samples when the original feature set is used. The DG GCL cluster identified with the refined feature set is present in all hippocampal samples (Fig. 1D) and is consistent with the expression of *CALB1*. Although application of BatchSVG did not substantially alter the NMI (slightly increasing from 0.51 to 0.52), the silhouette score range for the dentate gyrus molecular layer (DG ML) is greatly improved after refining the input SVG list with BatchSVG, as this corresponds to the biologically important segregation of the DG GCL from the ML (Fig. 1E; Fig. S4, available as supplementary data at *Bioinformatics* online). Finally, we note that common QC metrics do not differ between the DG GCL and ML domains from the original feature set versus the refined feature set (Fig. 1F). These results demonstrate the success of BatchSVG in reducing the batch-biased impact and enhancing biologically meaningful spatial domain identification.

### 3.2 Improved spatial domain detection in human dlPFC

This dataset contains human dorsolateral prefrontal cortex (dlPFC) tissue generated using Visium, with a total of twelve samples across three donors (Maynard *et al.* 2021). We chose three samples, one from each donor, and identified 1864 SVGs with nnSVG, specifically subsetting to genes that were statistically significant in at least two samples. We used BatchSVG with sample as the batch, nSD RCD as 4, and nSD RD as 7 to identify two genes as batch-biased SVGs (Fig. S5A and B, Table S2, available as supplementary data at *Bioinformatics* online). The PRECAST clustering results (*k *= 6 clusters) after using BatchSVG are substantially more consistent with known biology than the clusters based on the original feature set (Figs S5C and S6, available as supplementary data at *Bioinformatics* online). Specifically, the pink and green L3/4/5/6 domains in the pre-BatchSVG clustering result separate into two distinct domains expressing L3/4 and L5/6 marker genes, respectively. This improvement is quantified by the increase in NMI comparing either set of domains to manual annotations from 0.39 to 0.58. The silhouette scores for all domains are relatively similar before and after applying BatchSVG to the input SVG set (Figs S5D and S7, available as supplementary data at *Bioinformatics* online). We again observed that these changes were not associated with differences in QC metrics (Fig. S5E, available as supplementary data at *Bioinformatics* online).

## 4 Discussion

We developed BatchSVG as a method to identify genes in the SVG set that may be unwanted due to batch effects. We demonstrated that excluding these batch-biased SVGs improves downstream clustering in two SRT datasets and makes the domains more harmonious across samples. A brief sensitivity analysis shows the change in the number of batch-biased SVGs as the nSD of RCD and RD increases (Fig. S8, available as supplementary data at *Bioinformatics* online), which can be used along with the y=x lines in the svg_nSD() function to choose nSD thresholds. The goal of this method is not to replace batch effect correction and integration methods, but to help with interpretability by identifying genes associated with batch effects in SRT data. This method can be extended to imaging-based SRT data since gene-wise distributions are similar (Zhao *et al*. 2022), but future research would be necessary for validation.

## Supplementary Material

btag522_Supplementary_Data

## Data Availability

BatchSVG is based on the R programming language. This package is freely available at https://bioconductor.org/packages/devel/bioc/html/BatchSVG.html (Bioconductor version $>3.21),dependingonR(version>$ 4.4.0). All data analysis is freely available at https://github.com/kinnaryshah/BatchSVG-analyses or at https://doi.org/10.5281/zenodo.20737993.
