# Supplementary material for: BatchSVG: identifying batch-biased genes in the application of spatially variable gene detection
Source: Bioinformatics. 2026 Jul 16;42(7):btag522. doi: 10.1093/bioinformatics/btag522 (PMC13412158; doi:10.1093/bioinformatics/btag522)
Supplement: btag522_Supplementary_Data [file btag522_supplementary_data.pdf]

Supplementary Materials

BatchSVG: identifying biased features in application of spatially variable gene detection

Kinnary Shah, Christine Hou, Jacqueline R. Thompson, Stephanie C. Hicks*

*Correspondence to shicks19@jhu.edu

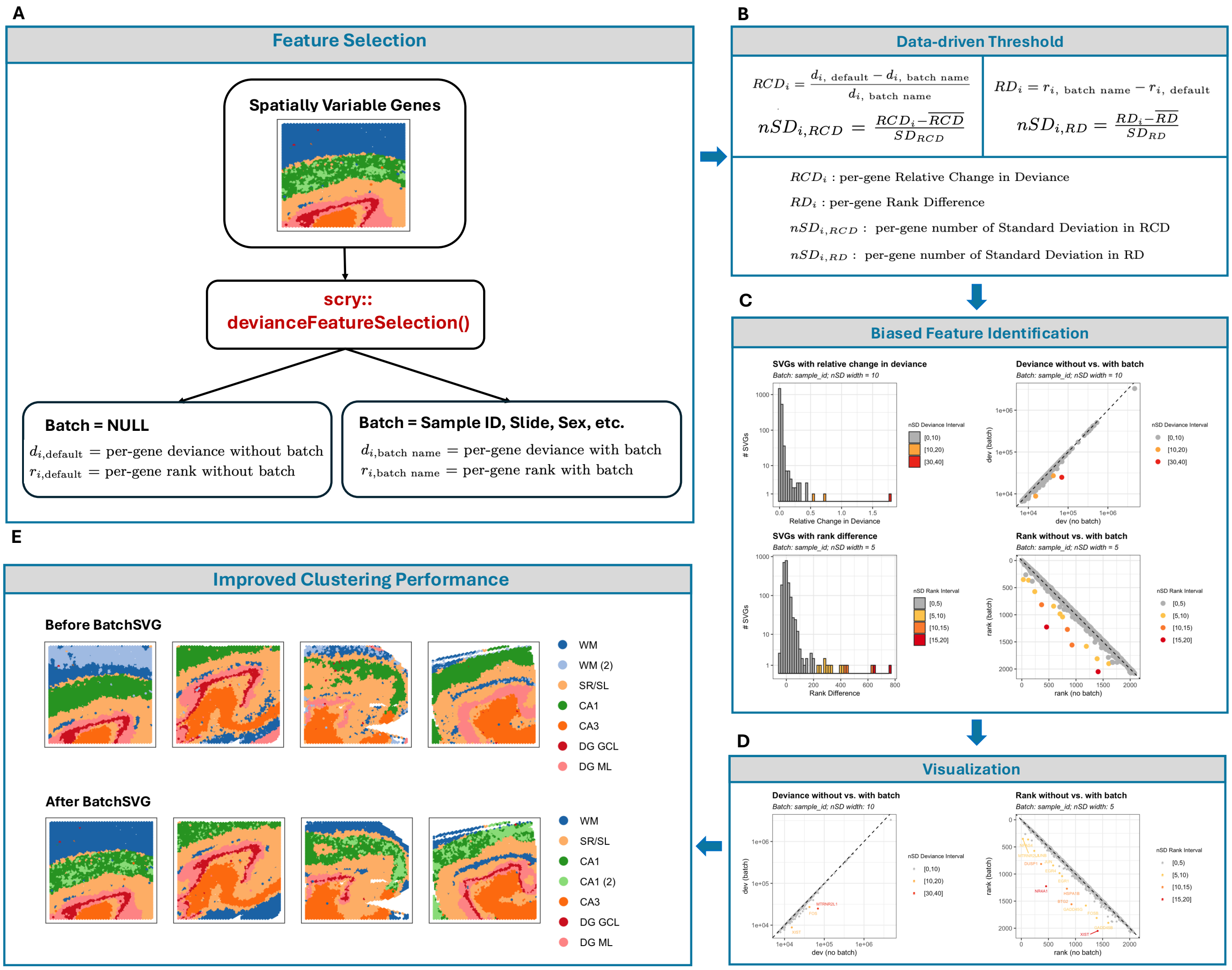

Figure S1: The standard workflow of `BatchSVG`: a feature-based quality control method to identify batch-biased spatially variable genes (SVGs). The data is the spatially resolved transcriptomics (SRT) human hippocampus dataset used in the main manuscript. (A) The binomial deviance model is applied to SVGs for per-gene deviance and rank with and without the selected batch variable(s). (B) Data-driven thresholds are computed as the per-gene number of Standard Deviation (nSD) for Relative Change in Deviance (RCD) and Rank Difference (RD). (C) The visualizations help determine the appropriate thresholds for nSD of RCD and RD. (D) `BatchSVG` can identify and visualize batch-biased features based on user-selected thresholds. (E) The refined SVG set, defined by removing batch-biased SVGs, can improve the downstream clustering performance. The clusters were annotated with the following labels - CA: cornu ammonis, DG GCL: dentate gyrus granule cell layer, DG ML: dentate gyrus molecular layer, SR/SL: stratum lucidum and stratum radiatum, WM: white matter.

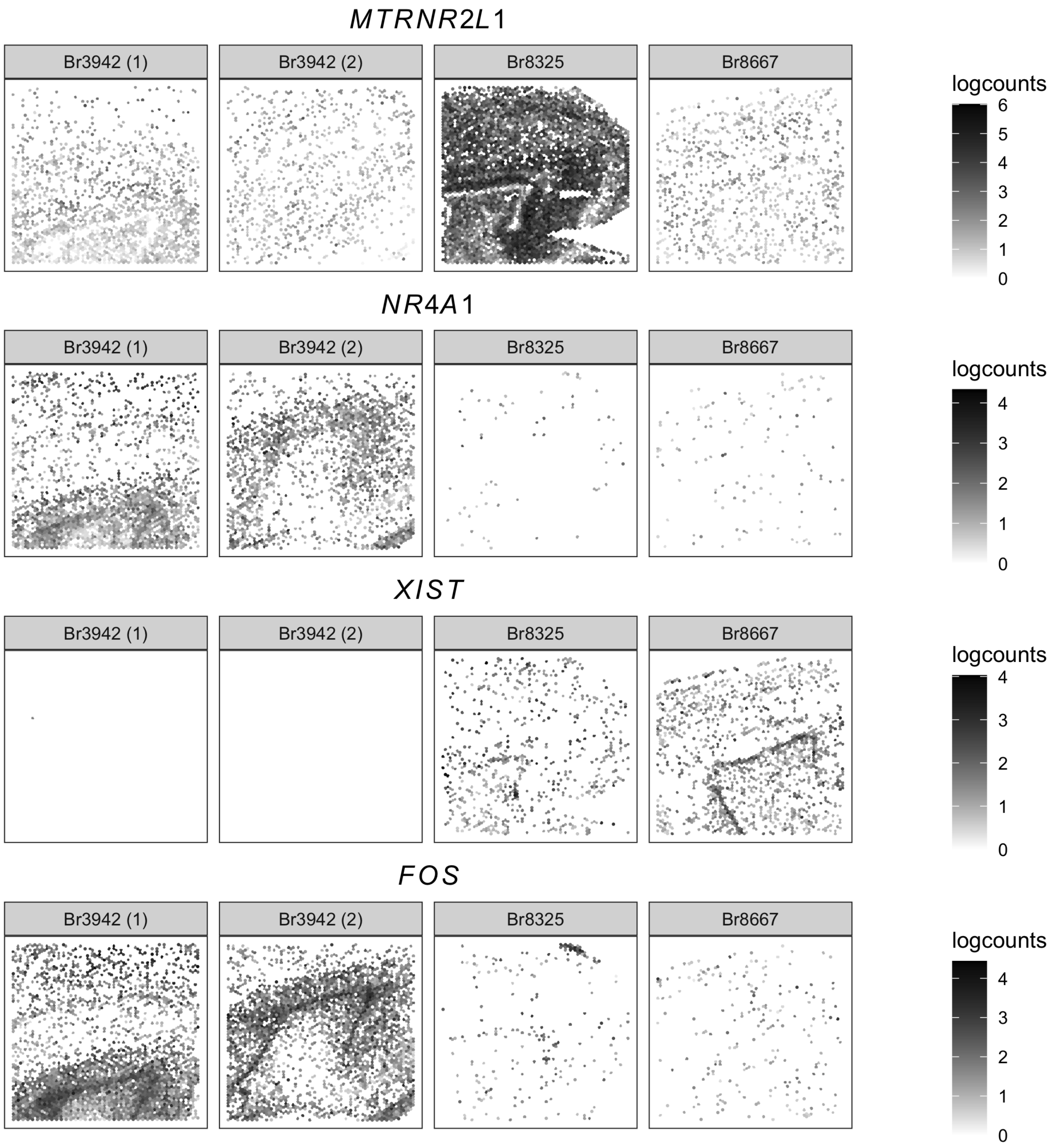

Figure S2: Selected spot plots of batch-biased SVGs found in the human hippocampus dataset. The gradient shows the log-normalized counts values for the following genes: *MTRNR2L1*, *NR4A1*, *XIST*, and *FOS*.

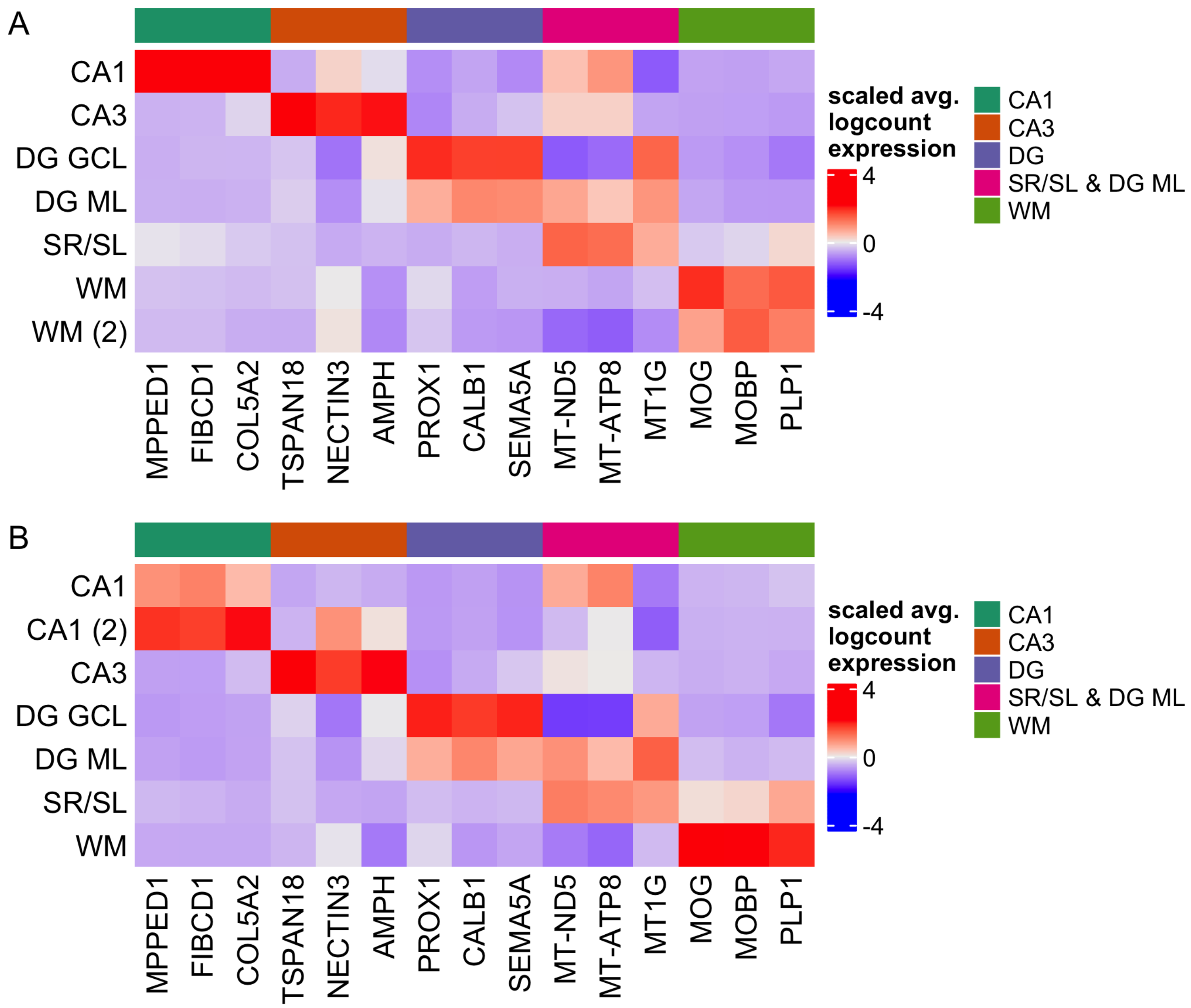

Figure S3: (A) Heatmap (centered and scaled) displays hippocampus regional marker genes for domains derived from `PRECAST` when all SVGs were used as input. The marker genes are associated with the following labels - CA: cornu ammonis, DG GCL: dentate gyrus granule cell layer, DG ML: dentate gyrus molecular layer, SR/SL: stratum lucidum and stratum radiatum, WM: white matter. (B) Heatmap (centered and scaled) displays hippocampus regional marker genes for domains derived from `PRECAST` when the batch-biased SVGs were excluded from the input SVG list. The marker genes are associated with the following labels - CA: cornu ammonis, DG GCL: dentate gyrus granule cell layer, DG ML: dentate gyrus molecular layer, SR/SL: stratum lucidum and stratum radiatum, WM: white matter.

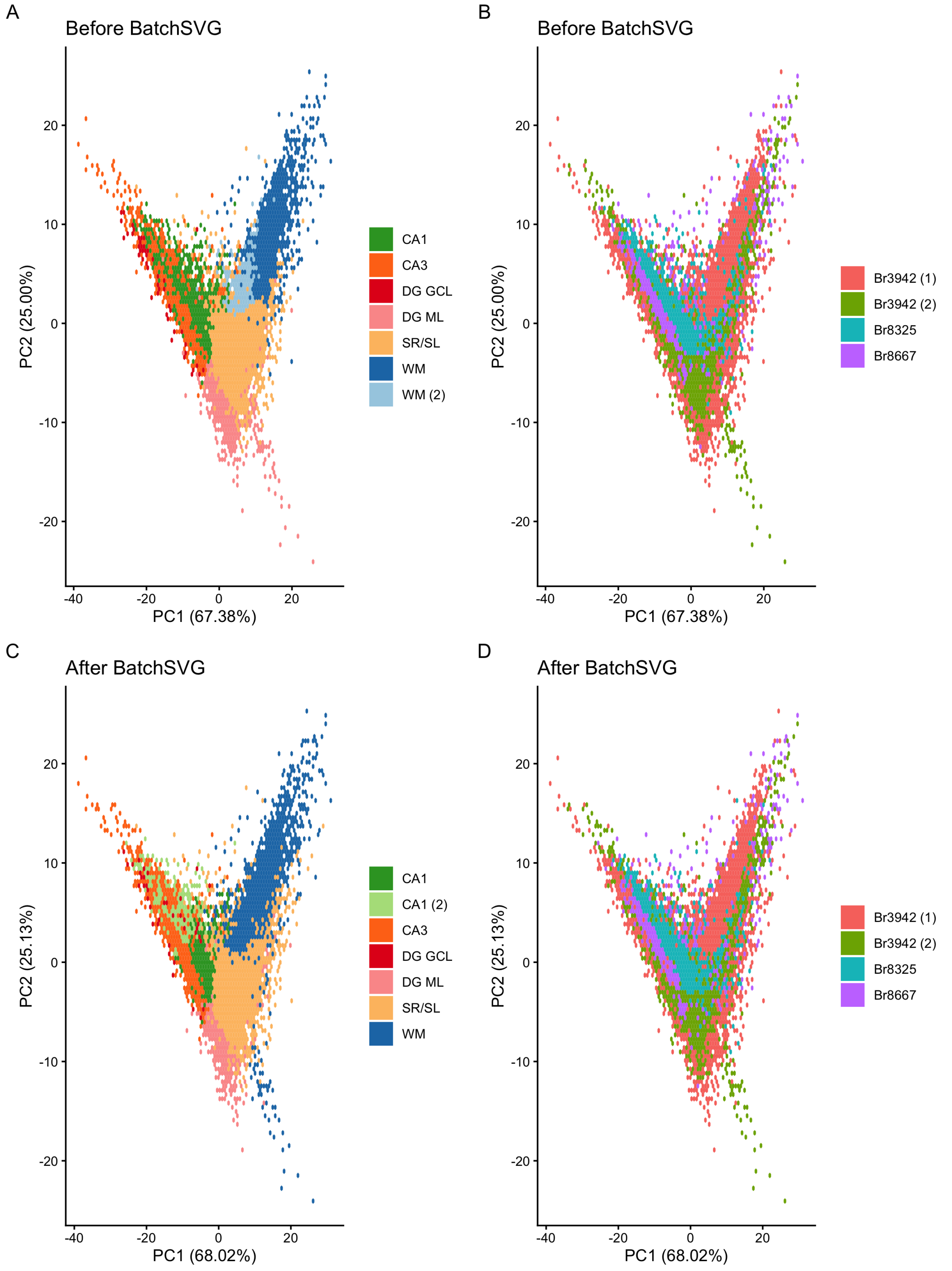

Figure S4: Principal components analysis (PCA) plots of the the human hippocampus dataset. (A and B) PCA plots of the first two principal components (PCs) for the domains derived from `PRECAST` when all SVGs were used as input. (C and D) PCA plots of the first two PCs for the domains derived from `PRECAST` when the batch-biased SVGs were excluded from the input SVG list. Color of each hexbin represents the majority cluster. The clusters were annotated with the following labels - (A and C) CA: cornu ammonis, DG GCL: dentate gyrus granule cell layer, DG ML: dentate gyrus molecular layer, SR/SL: stratum lucidum and stratum radiatum, WM: white matter. (B and D) sample name.

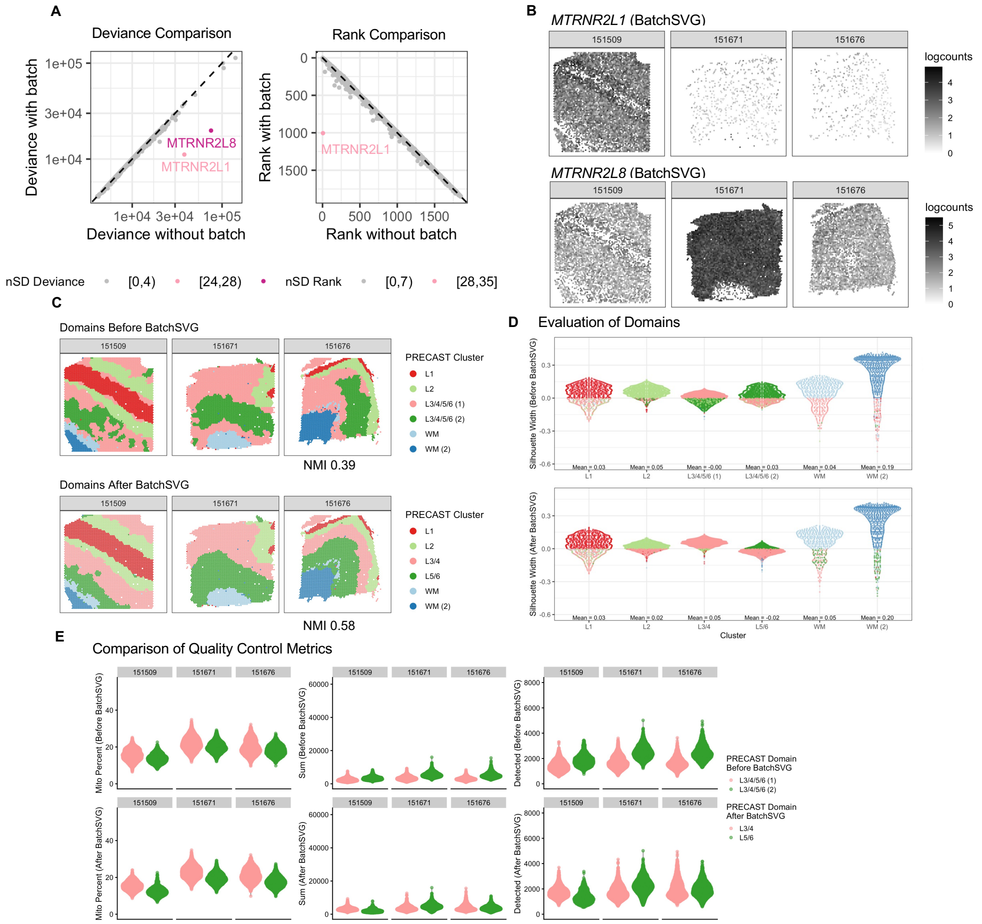

Figure S5: Summary figure for dorsolateral prefrontal cortex dataset. (A) Left scatterplot displays the change in deviance for each gene. The x-axis is deviance calculated without using any covariates. The y-axis is deviance calculated with sample as the covariate. The color represents the number of standard deviations of relative change in deviance. Right scatterplot is similar, substituting rank for deviance. (B) Top spot plot shows the log-normalized counts gene expression for *MTRNR2L1*, which is a batch-biased SVG. Bottom spot plot shows the log-normalized counts gene expression for *MTRNR2L8*, another batch-biased SVG. (C) Top spot plot shows the clusters derived from `PRECAST` when all SVGs were used as input. Bottom spot plot shows the clusters derived from `PRECAST` when the batch-biased SVGs were excluded from the input SVG list. The clusters were annotated with the following labels - L + number: Layer number, WM: white matter. Color represents the cluster. Text displays the normalized mutual information (NMI) for each set of domains compared to manual annotations. (D) Top violin plots display silhouette scores for domains derived from `PRECAST` when all SVGs were used as input. Bottom violin plots display silhouette scores for domains derived from `PRECAST` when the batch-biased SVGs were excluded from the input SVG list. Color represents the closest domain. (E) Violin plots display various quality control metrics for domains derived from `PRECAST` when the batch-biased SVGs were included (top row) or excluded (bottom row) from the input SVG list. Left row displays mitochondrial percent, middle row displays total number of UMIs, and right row displays number of detected genes. Color represents the domain.

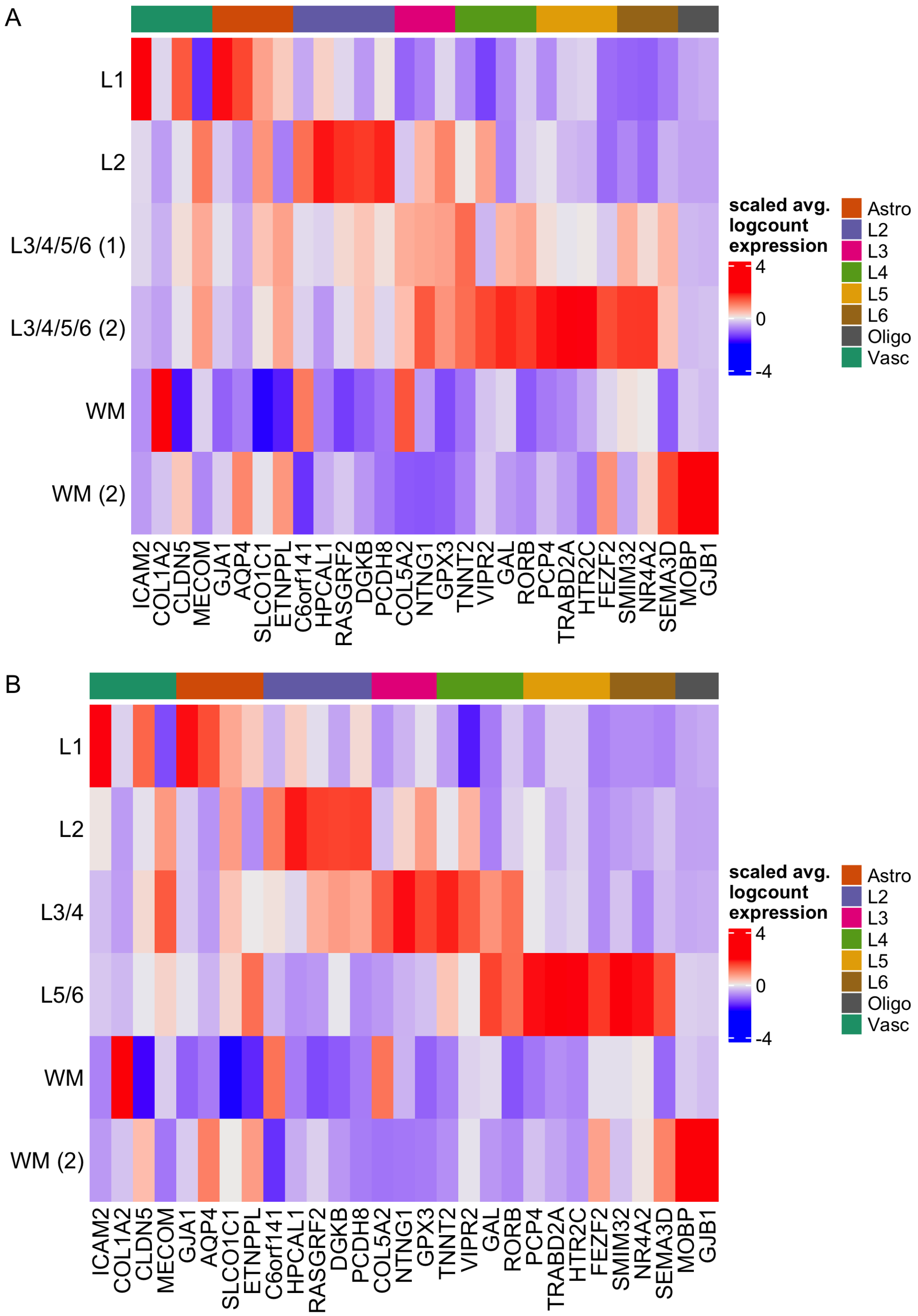

Figure S6: (A) Heatmap (centered and scaled) displays dorsolateral prefrontal cortex laminar marker genes for domains derived from `PRECAST` when all SVGs were used as input. The marker genes are associated with the following labels - Astro: astrocyte, L + number: Layer number, Oligo: oligodendrocyte, Vasc: vasculature. (B) Heatmap (centered and scaled) displays dorsolateral prefrontal cortex laminar marker genes for domains derived from `PRECAST` when the batch-biased SVGs were excluded from the input SVG list. The marker genes are associated with the following labels - Astro: astrocyte, L + number: Layer number, Oligo: oligodendrocyte, Vasc: vasculature.

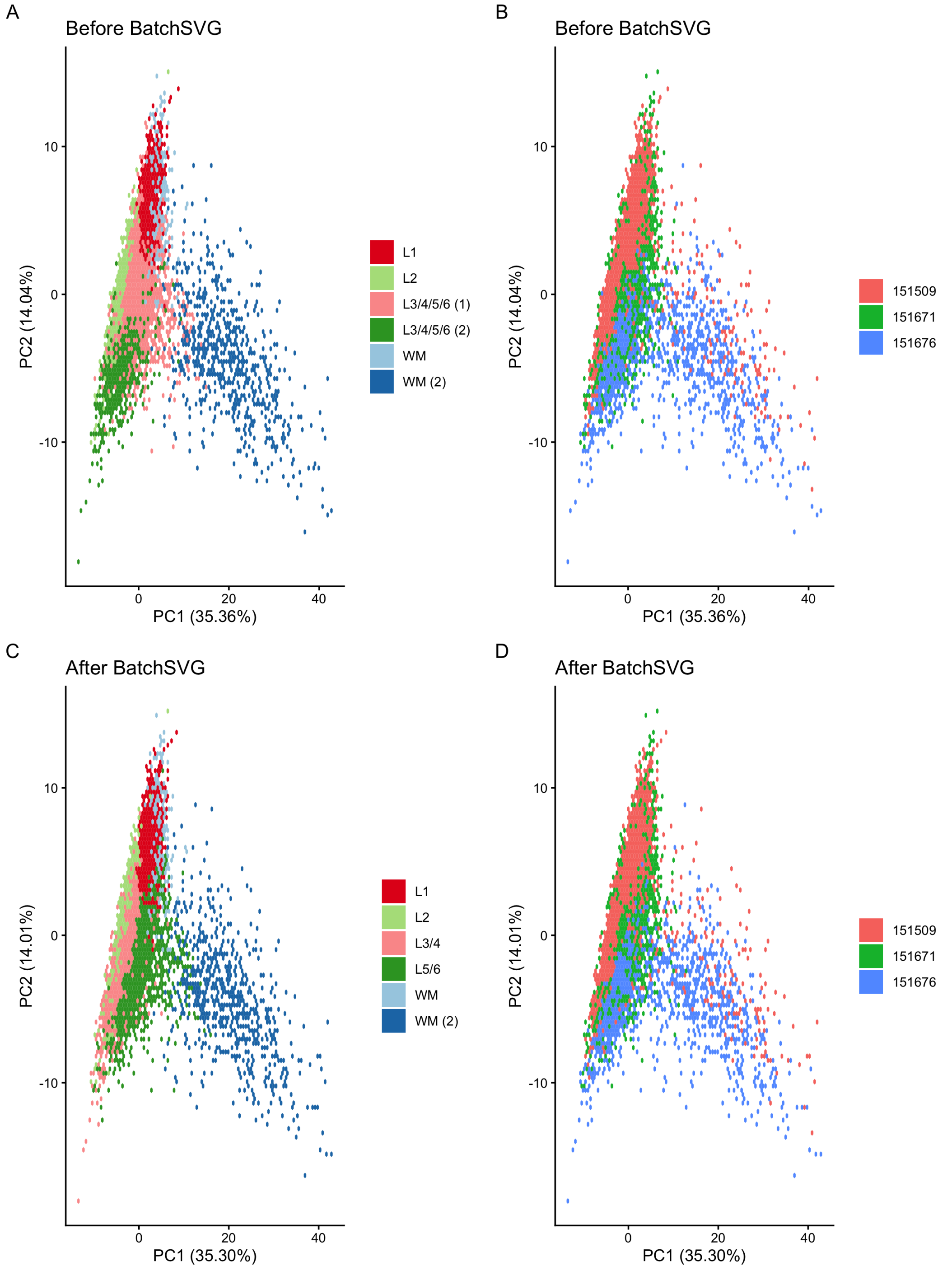

Figure S7: Principal components analysis (PCA) plots of the human dorsolateral prefrontal cortex dataset. (A and B) PCA plots of the first two principal components (PCs) for the domains derived from `PRECAST` when all SVGs were used as input. (C and D) PCA plots of the first two PCs for the domains derived from `PRECAST` when the batch-biased SVGs were excluded from the input SVG list. Color of each hexbin represents the majority cluster. The clusters were annotated with the following labels - (A and C) L + number: Layer number, WM: white matter. (B and D) sample name.

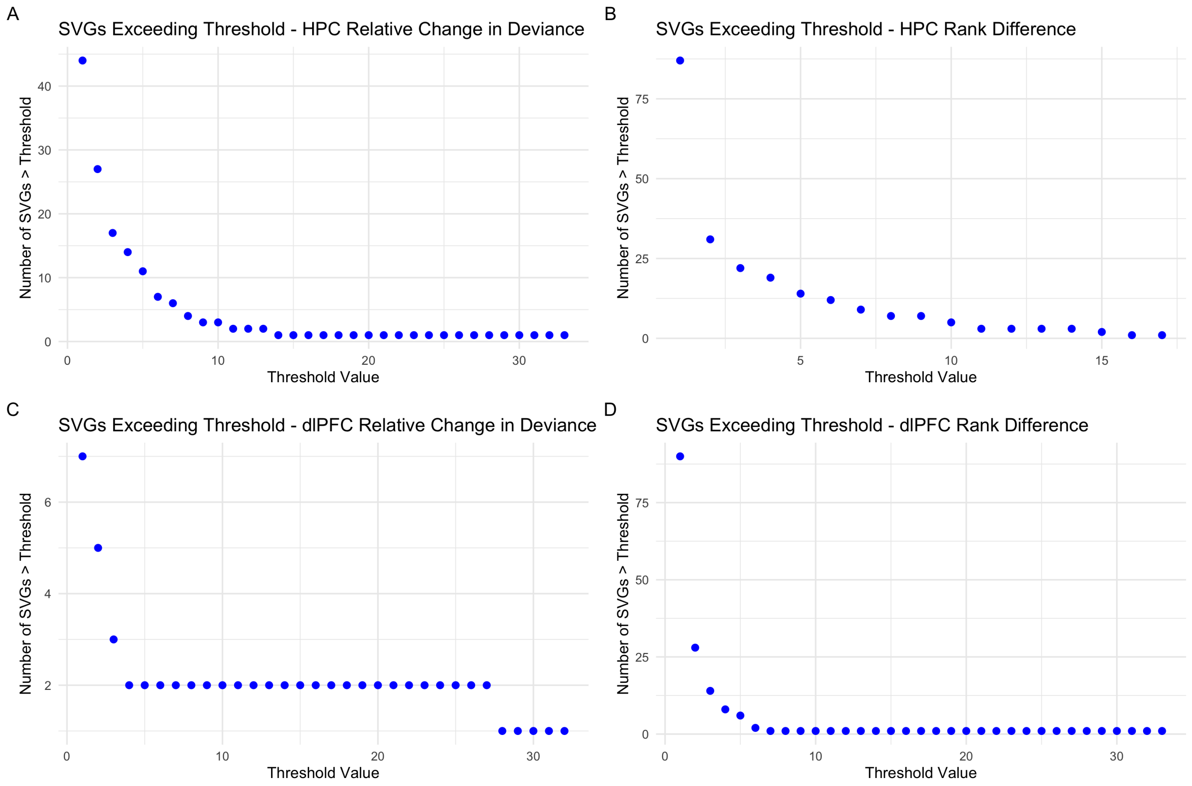

Figure S8: Sensitivity analyses for nSD cutoff thresholds. (A) Scatterplot displays the variation in the number of batch-biased SVGs based on the choice of nSD of RCD thresholds for the human hippocampus dataset, where the x-axis is the nSD of RCD and the y-axis is the number of batch-biased SVGs when using that threshold. (B) Similar to (A) for the choice of nSD of RD thresholds for the human hippocampus dataset. (C) Similar to (A) for the choice of nSD of RCD thresholds for the human dorsolateral prefrontal cortex dataset. (D) Similar to (A) for the choice of nSD of RD thresholds for the human dorsolateral prefrontal cortex dataset.

Table S1: Human hippocampus batch-biased SVGs. This table shows all genes found to be batch-biased SVGs from the human hippocampus dataset.

Gene Name	Gene ID
JUN	ENSG00000177606
BTG2	ENSG00000159388
EGR4	ENSG00000135625
EGR1	ENSG00000120738
DUSP1	ENSG00000120129
HSPA1B	ENSG00000204388
GADD45G	ENSG00000130222
NPAS4	ENSG00000174576
NR4A1	ENSG00000123358
FOS	ENSG00000170345
MTRNR2L1	ENSG00000256618
GADD45B	ENSG00000099860
JUNB	ENSG00000171223
FOSB	ENSG00000125740
XIST	ENSG00000229807

Table S2: Human dorsolateral prefrontal cortex batch-biased SVGs. This table shows all genes found to be batch-biased SVGs from the human dorsolateral prefrontal cortex dataset.

Gene Name	Gene ID
MTRNR2L1	ENSG00000256618
MTRNR2L8	ENSG00000255823
